# Long-term changes in renal function and perfusion in heart failure patients with reduced ejection fraction

**DOI:** 10.1007/s00392-015-0881-9

**Published:** 2015-06-30

**Authors:** Nicolas F. Schroten, Kevin Damman, Mattia A. E. Valente, Tom D. Smilde, Dirk J. van Veldhuisen, Gerjan Navis, Carlo A. Gaillard, Adriaan A. Voors, Hans L. Hillege

**Affiliations:** Department of Cardiology, University Medical Center Groningen, Groningen, The Netherlands; Department of Cardiology, Scheper Ziekenhuis, Emmen, The Netherlands; Department of Internal Medicine, University Medical Center Groningen, Groningen, The Netherlands; Department of Epidemiology, University Medical Center Groningen, Hanzeplein 1, 9713GZ Groningen, The Netherlands

**Keywords:** Cardiorenal, Heart failure, Renal blood flow, Kidney, Biomarkers

## Abstract

**Introduction:**

Little is known about the natural course of renal function and renal hemodynamics in heart failure patients with reduced ejection fraction (HFREF).

**Methods and results:**

We prospectively studied effective renal plasma flow (ERPF) and glomerular filtration rate (GFR) in 73 HFREF patients with ^125^I-iothalamate/^131^I-hippuran clearances with a mean follow-up of 34.6 ± 4.4 months. Fifteen percent were female, with age 58 ± 12 years and left ventricular ejection fraction (LVEF) 29 ± 10 %. Baseline GFR was 81 ± 23 mL/min/1.73 m^2^ and declined 0.6 ± 4.7 mL/min/1.73 m^2^ per year. Baseline ERPF was 292 ± 83 mL/min/1.73 m^2^ and declined 4.3 ± 19 mL/min/1.73 m^2^ per year. Of the baseline variables, older age and high urinary kidney injury molecule-1 were the only variables associated with GFR decline (*p* < 0.05). Following stepwise backward analysis, only age (*p* < 0.001) remained significant. In addition, we found an association between change in GFR and changes in ERPF, *N*-terminal pro-brain natriuretic peptide and renovascular resistance. In the multivariable analysis, only the change in ERPF remained significantly associated with a change in GFR (*p* < 0.001).

**Conclusion:**

In this cohort of stable chronic HFREF patients, the average decline in GFR over time was small. The decline of GFR was associated with a higher age and a lower baseline GFR, and was strongly related to changes in renal perfusion.

**Electronic supplementary material:**

The online version of this article (doi:10.1007/s00392-015-0881-9) contains supplementary material, which is available to authorized users.

## Introduction

Both chronic kidney disease (CKD) and worsening renal function are common in heart failure patients [1–3] and among the most powerful predictors of morbidity and mortality in this population [4]. However, little is known about the natural course of renal function in heart failure patients and determinants of long-term renal function decline. The cause of renal dysfunction in HFREF is thought to be multifactorial [5, 6]. It has been attributed to medication [7], renin–angiotensin–aldosterone system (RAAS) activation [8], sympathetic nervous system (SNS) activation and inflammation. Decreased renal perfusion is likely the key determinant [9], via decreased renal perfusion pressure, an increase in renovascular resistance (RVR), increase in renal venous pressure or all of the above [10]. However, these associations have mostly been described in cross-sectional studies. The limited number of longitudinal studies has mostly focused on acute worsening of renal function, and few data are available on predictors of long-term estimated glomerular filtration rate (GFR) changes in heart failure patients with reduced ejection fraction (HFREF) [11–14]. All these studies used changes in serum creatinine to estimate GFR, which is considered a surrogate for the functioning kidney tissue. However, creatinine-based renal function estimates are not always accurate in estimating kidney function decline [15] and provide no information on renal hemodynamics.

Using gold standard techniques for measuring renal function, we studied the change in renal function over time and its clinical, biochemical and hemodynamic predictors in patients with heart failure. We previously described the cross-sectional associations. Renal blood flow showed the strongest association with GFR. In turn, *N*-terminal pro-brain natriuretic peptide (NT-proBNP), plasma renin activity, soluble vascular cell adhesion molecule-1 (sVCAM-1) levels and urinary albumin excretion (UAE) showed the strongest associations with renal blood flow [9]. In the current analysis, we investigated if these parameters are also associated with long-term renal function decline, measured using radioactive labeled specific renal function tracers.

## Methods

### Patient population

Details on the study design and patient population have been published previously [9]. In brief, 120 clinically stable HFREF patients, with left ventricular ejection fraction (LVEF) <45 % and stable heart failure medication for at least 1 month underwent renal function measurements using ^125^I-iothalamate and ^131^I-hippuran clearance techniques at the University Medical Center Groningen, The Netherlands. Blood and urine samples were collected, a physical examination performed and the patient’s history documented. Patients were contacted after 3 years and all investigations were repeated. The study was approved by the ethics committee of the study center, and all subjects gave written informed consent. The study was conducted in accordance with Declaration of Helsinki guidelines.

### Renal and cardiac function measurements

Renal function measurements were performed using radioactive labeled tracers, ^125^I-iothalamate and ^131^I-hippuran, as described previously [16]. This method has an intra- and inter-test variation of 1.9 and 2.9 %, respectively, for GFR. The intra-subject day-to-day CV of effective renal plasma flow (ERPF) is 5.0 % [17]. The filtration fraction was calculated as GFR/ERPF. RVR was calculated as (mean arterial pressure/ERPF) × (1 − hematocrit) and expressed in mmHg/mL/min. GFR and ERPF were corrected for 1.73 m^2^ of body surface area, calculated using the Dubois formula. LVEF was determined by nuclear ventriculography.

### Laboratory methods

Patients were all in the supine position during renal measurements, and a venous blood sample was drawn 2 h after the start of the measurements. Routine hematology, blood chemistry and urinalysis were performed within an hour of collection. Additional blood and urine samples were immediately centrifuged and stored at −80 °C. Urinary markers of renal damage were measured in 24 h urine collections and corrected for urinary creatinine as described previously [18]. A detailed description of the methods and analytical variation is provided in supplement 1.

### Follow-up

All patients were asked to return for a follow-up visit between 24 and 36 months after baseline renal function measurements. All measurements performed at baseline were repeated including laboratory analyses, renal function measurements using radioactive labeled tracers and nuclear ventriculography. Adverse events during follow-up were determined via interview and case record extraction. Adverse events included death from any cause, heart transplantation, cardiovascular event (myocardial infarction or primary percutaneous coronary intervention or primary coronary artery bypass grafting) and first hospitalization for worsening heart failure.

### Statistical analyses

Continuous data are presented as mean ± standard deviation (SD) when normally distributed, as median and inter-quartile range when non-normally distributed and as frequencies and percentages for categorical variables. Differences between groups were tested using Student’s *T* test, Kruskal–Wallis or Chi-square test as appropriate. Linear regression analysis was carried out to determine the association of baseline variables with change in GFR and to test the association of changes in hemodynamic parameters with changes in GFR. Linear regression models with delta variables were corrected for baseline values of the variables of interest. Age and sex were included in all multivariable models. Skewed variables were log-transformed where appropriate. Variables associated with the univariable model at *p* < 0.1 were included in a stepwise, backward multivariable regression analysis, with a threshold for variable retention of *p* < 0.1. All reported probability values are two tailed, and a *p* value of <0.05 was considered to be statistically significant. Statistical analyses were performed and graphics created using STATA version 11.0, College Station, TX, USA.

## Results

**Fig. 1:**
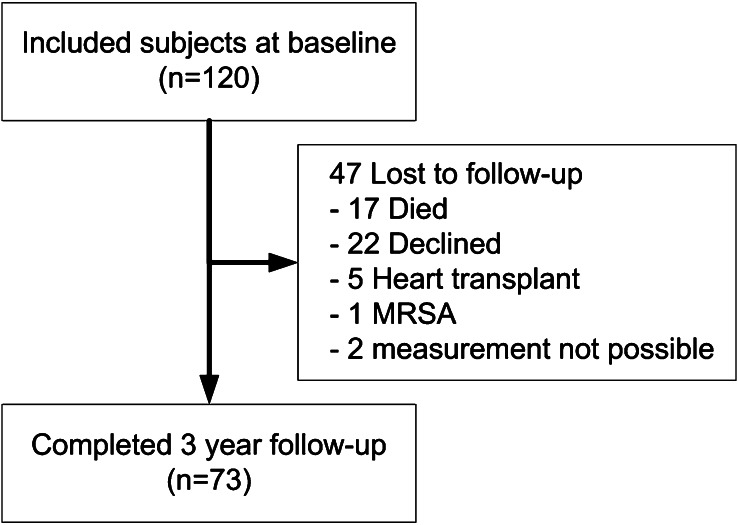
Patient disposition

Of the 120 patients included at baseline, 73 returned for follow-up measurements (Fig. 1). The baseline characteristics of the study population are presented in Table 1. In brief, 15 % were female, with a mean age of 58 ± 12 years. The left ventricular ejection fraction (LVEF) was 29 ± 10 %. Most patients had New York Heart Association (NYHA) class II or III heart failure symptoms. All patients were on an angiotensin-converting enzyme inhibitor and/or angiotensin receptor blocker and most were on beta-blocker therapy or aldosterone receptor antagonists.

**Table 1:** Baseline characteristics

Variable	With follow-up	Lost to follow-up	Died/HTX
	(*n* = 73)	(*n* = 25)	(*n* = 22)
Age (years)	58 ± 12	58 ± 12	62 ± 12
Female sex, *n* (%)	11 (15 %)	7 (28 %)	6 (27 %)
RR systolic (mmHg)	121 ± 18	127 ± 21	105 ± 20^#^
RR diastolic (mmHg)	71 ± 11	70 ± 11.2	62 ± 11^#^
Heart rate (bpm)	64 ± 12	66 ± 12	68 ± 16
Ischemic etiology, *n* (%)	39 (53 %)	11 (44 %)	10 (46 %)
LVEF (%)	29 ± 10	30 ± 10	28 ± 10
GFR (mL/min/1.73 m^2^)	81 ± 23	75 ± 28	50 ± 26^#^
ERPF (mL/min/1.73 m^2^)	292 ± 83	264 ± 90	202 ± 73^#^
Filtration fraction (%)	28 ± 3	28 ± 6	24 ± 8^#^
RVR (mmHg/mL/min)*	0.17 (0.15–0.22)	0.19 (0.14–0.29)	0.22 (0.18–0.31)^#^
UAE (mg/24 h)*	8 (6–12)	12 (7–35)	18 (7–49)^#^
NT-proBNP (ng/mL)*	465 (219–1100)	635 (286–1700)	2200 (950–5000)^#^
Urine NGAL (μg/24 h)*	15 (7–31)	17 (11–34)	9 (2–33)
Urine KIM-1 (U/24 h)*	408 (144–995)	416 (111–1800)	279 (20–1100)
Urine NAG (ng/24 h)*	4.4 (2.2–6.6)	3.7 (2.5–7.9)	3.4 (2.4–7.5)
ACE inhibitor, *n* (%)	65 (89 %)	21 (84 %)	16 (73 %)
ARB, *n* (%)	8 (11 %)	3 (12 %)	7 (32 %)**
Beta-blocker, *n* (%)	63 (86 %)	20 (80 %)	18 (82 %)
Aldosterone antagonist, *n* (%)	18 (25 %)	6 (24 %)	13 (59 %)^#^

Normally distributed data are presented as mean ± SD; * skewed data as median (p25–p75)

*RR* blood pressure, *LVEF* left ventricular ejection fraction, *GFR* glomerular filtration rate, *ERPF* effective renal plasma flow, *RVR* renovascular resistance, *UAE* urinary albumin excretion, *NT-proBNP* *N*-terminal pro-brain natriuretic peptide, *NGAL* neutrophil gelatinase-associated lipocalin, *KIM-1* kidney injury molecule 1, *NAG* *N*-acetyl-b-d-glucosaminidase, *ARB* angiotensin receptor blocker, *ACE* angiotensin-converting enzyme

** *p* < 0.05 and ^#^ p < 0.01 compared with patients with complete follow-up

Baseline GFR was 81 ± 23 mL/min/1.73 m^2^ and baseline ERPF was 292 ± 83 mL/min/1.73 m^2^. The mean follow-up time was 34.6 ± 4.4 months. In patients with a complete follow-up, the mean decline in GFR was 0.6 ± 4.7 ml/min/1.73 m^2^ per year and ERPF declined 4.3 ± 19 mL/min/1.73 m^2^ per year. There was no significant difference in the rate of renal function decline between patients with a GFR below and above 60 mL/min/1.73 m^2^ at baseline (*p* = 0.81). Patients who were lost to follow-up are also presented in Table 1. Patient who died or had a heart transplant during follow-up had a lower blood pressure, GFR, ERPF and filtration fraction, and a higher RVR, UAE and NT-proBNP and were more often using angiotensin receptor blockers (ARB) or aldosterone receptor antagonists (ARA) compared with patients who completed follow-up. There were no significant differences between patients who completed follow-up and those who were lost to follow-up for other reasons.

### Predictors of changes in GFR

#### Baseline variables

Associations of baseline characteristics and laboratory tests with change in GFR are shown in Table 2. Baseline age, sex, mean arterial pressure, neutrophil gelatinase-associated lipocalin (NGAL) and kidney injury molecule 1 (KIM-1) showed a relation with change in GFR at *p* < 0.1 (Table 2). Following stepwise backward analysis, only older age (*p* < 0.001) remained significantly associated with higher GFR decline in a multivariable model.

**Table 2:** Association of baseline markers with GFR change (mL/min/1.73 m^2^) per year corrected for baseline GFR, age and sex

Variable	Coef	95 % CI	Beta	*p* value
Age (years)	−0.25	(−0.34 to −0.17)	−0.64	<0.001
Female sex	2.7	(−0.31 to 5.75)	0.21	0.077
MAP (mmHg)	−0.08	(−0.17 to 0.01)	−0.22	0.064
LVEF (%)	−0.08	(−0.21 to 0.04)	−0.17	0.20
ERPF (mL/min/1.73 m^2^)	0.01	(−0.02 to 0.04)	0.17	0.54
RVR (mmHg/mL/min)	−7.9	(−27 to 9.9)	−0.15	0.38
Filtration fraction (%)	−0.09	(−0.42 to 0.24)	−0.06	0.61
NT-proBNP (ng/mL)*	−0.29	(−0.98 to 0.40)	−0.11	0.41
Hemoglobin (mmol/l)	0.15	(−1.6 to 1.9)	−0.02	0.87
CRP (mg/L)*	−0.17	(−0.93 to 0.60)	−0.05	0.67
24 h Urine sodium (mmol)	−4.6	(−14 to 4.8)	−0.44	0.29
UAE (mg/24 h)*	−0.19	(−0.79 to 0.41)	−0.08	0.53
NGAL (μg/24 h)*	−0.60	(−1.3 to 0.11)	−0.21	0.096
KIM-1 (U/24 h)*	−0.55	(−1.0 to −0.7)	−0.27	0.027
NAG (ng/24 h)*	−0.34	(−1.1 to 0.41)	−0.11	0.36

*GFR* glomerular filtration rate, *MAP* mean arterial pressure, *LVEF* left ventricular ejection fraction, *ERPF* effective renal plasma flow, *RVR* renovascular resistance, *NT-proBNP* *N*-terminal pro-brain natriuretic peptide, *CRP* C-reactive protein, *UAE* urinary albumin excretion, *NGAL* neutrophil gelatinase-associated lipocalin, *KIM-1* kidney injury molecule 1, *NAG* *N*-acetyl-b-d-glucosaminidase

* log-transformed variables

#### Changes in hemodynamics and renal perfusion

**Fig. 2:**
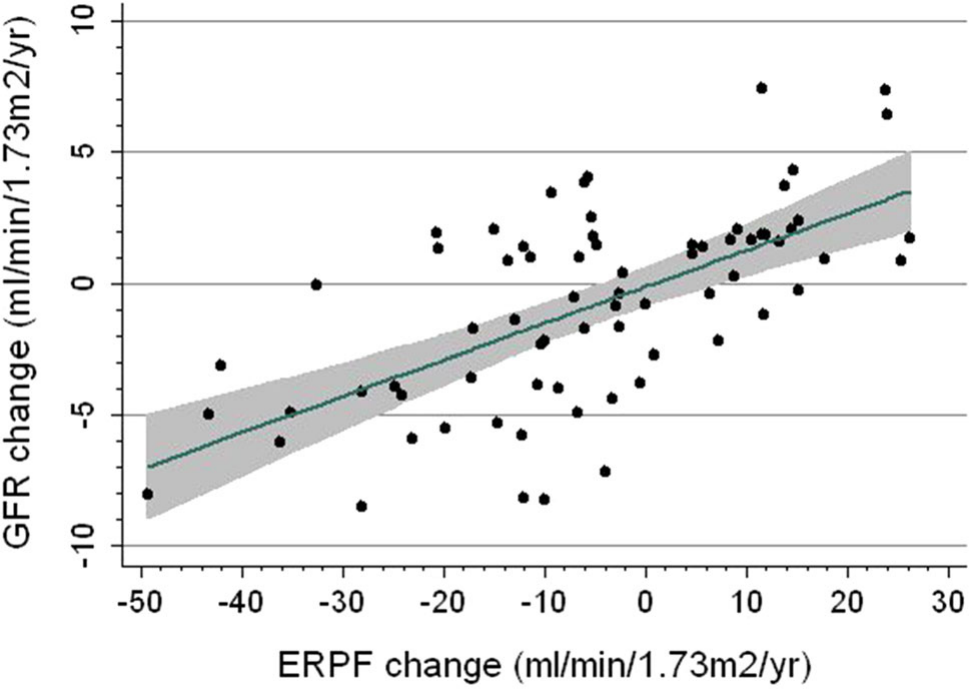
Changes in GFR and ERPF

In general, patients who completed follow-up maintained a relatively stable hemodynamic profile. Changes in LVEF (+3.3 ± 11 %), mean arterial pressure (−0.13 ± 10 mmHg), NT-proBNP [−0.6 (−265 to +250.6)ng/L] and RVR (0.01 ± 0.05 mmHg/mL/min) were modest. A decrease in ERPF and NT-proBNP and increase in RVR were associated with a decrease in GFR, while LVEF was not (Table 3; Fig. 2). In the multivariable analysis, only change in ERPF remained significantly associated with a change in GFR. In parallel to changes in GFR, an increase in RVR and a decrease in NT-proBNP and LVEF were associated with a decrease in ERPF. In multivariable analysis, only RVR and NT-proBNP remained significantly associated with changes in ERPF (results not shown). Change in mean arterial pressure was not associated with a change in either GFR or ERPF.

**Table 3:** Associations between hemodynamic changes and changes in GFR

	Coef	95 % CI	Beta	*p* value
Delta MAP (mmHg)	−0.17	(−0.47 to 0.13)	−0.14	0.25
Delta LVEF (%)	0.20	(−0.08 to 0.47)	0.16	0.16
Delta ERPF (mL/min/1.73 m^2^)	0.15	(0.11 to 0.19)	0.62	<0.001
Delta RVR (mmHg/mL/min)	−110	(−172 to −47.9)	−0.43	0.001
Delta log-NT-proBNP (ng/mL)	3.20	(1.17 to 5.22)	0.34	0.002

Models include age, sex and baseline values of the variable of interest

*GFR* glomerular filtration rate, *MAP* mean arterial pressure, *LVEF* left ventricular ejection fraction, *ERPF* effective renal plasma flow, *RVR* renovascular resistance, *NT-proBNP* *N*-terminal pro-brain natriuretic peptide

## Discussion

In the present study of patients with stable HFREF, we found only a small decrease in GFR over a longer period of time, in the order of magnitude also reported as the age-related decline in the general population. Likewise, ERPF decline did not differ much from the age-related decline rate in the general population [19]. Change in GFR was strongly associated with a parallel change in ERPF. Only higher age and lower baseline GFR predicted a greater decline in GFR over time, but none of the tested urinary biomarkers of renal damage or hemodynamic parameters were associated with GFR decline.

Several studies have focused on markers predicting worsening renal function in chronic heart failure, with limited success. The identified risk factors include congestion [20], vascular disease, diuretics, advanced age, left ventricular ejection fraction and worse renal function at baseline [4, 7, 11]. Furthermore, NGAL and NT-proBNP have been linked to worsening renal function in acute heart failure [21–23] and chronic heart failure [24]. However, all these studies used plasma creatinine to estimate GFR and cannot differentiate between changes in hemodynamics and kidney damage. In a previous analysis we demonstrated a strong relation of renal blood flow with GFR in HFREF patients [9].

In the current analysis, we found that none of the urinary biomarkers or hemodynamic parameters at baseline could predict renal function decline. Our study may have limited power, because of the small change in GFR over time; however, most of the aforementioned studies also demonstrated a limited estimated GFR decline over time and by using radioactive labeled tracers we can measure small changes in GFR more accurately. We cannot exclude that deceased subjects had a more rapid renal function decline. These subjects did have a lower GFR and ERPF and higher NT-proBNP at baseline; however, tubular damage markers were not elevated in these subjects. What is most remarkable is that they had a high RVR in combination with a low filtration fraction and low blood pressure. This may reflect the kidneys’ inability to maintain glomerular perfusion pressure. They were more often on double renin–angiotensin–aldosterone system (RAAS) blockers, which may decrease the filtration fraction by vasodilation of the efferent glomerular arteriole; however, this should cause a decrease in RVR. The high RVR, therefore, must reflect a different mechanism, possibly compromised kidney perfusion by increased venous pressure, sympathetic nerve activation or a decreased amount of functioning glomeruli.

In our study, we found that the change in ERPF was the strongest determinant of the change in GFR. In contrast, in healthy individuals, GFR remains relatively stable with moderate changes in renal blood flow [25]. It may be speculated that impaired systemic circulation causes decreased ERPF and, because of impaired intra-renal regulatory mechanisms, a parallel decline in GFR, but it may also imply that both ERPF and GFR are affected by intrarenal hemodynamic changes. Both congestion and reduced cardiac output are thought to influence renal function in heart failure patients. In our study, an increase in NT-proBNP was associated with an increase in ERPF and GFR. This is counterintuitive, since higher NT-proBNP is associated with worsening cardiac function [26]. However, changes in volume status also influence NT-proBNP levels, suggesting that not only congestion, but also hypovolemia causes renal function decline in these patients. Another explanation for the observed relationship is that kidney damage affects both ERPF and GFR. However, many patients showed an increase in ERPF and an associated increase in GFR, which suggests changes in hemodynamics rather than in viable kidney tissue.

This study has several limitations. First, not all patients were able to participate in the second measurement. The deceased patients had worse baseline renal function, lower blood pressure and higher NT-proBNP. Second, we only had two measurements; therefore, we cannot establish if there is a linear trend over time and cannot account for fluctuations. Furthermore, our study has a modest sample size. The measurements performed, however, are the gold standard for measuring renal function, with a day-to-day variation coefficient of less than 3 % for GFR and 5 % for ERPF. Patients were mostly stable on medication; however, some patients had minor changes in dose or type of medication. Finally, this was a relatively young cohort, with mostly male Caucasian patients.

## Conclusion

In these stable chronic HFREF patients, long-term changes in GFR were small, but strongly related to changes in ERPF. None of the investigated urinary biomarkers and hemodynamic parameters other than baseline GFR and age could predict changes in GFR. This underlines the need for the development of new renal risk markers and demonstrates that changes in GFR are mostly driven by changes in renal hemodynamics in chronic HFREF patients. Intervention trials should investigate whether targeting ERPF may improve GFR and reduce cardiac events and mortality.

## Electronic supplementary material

Supplementary material 1 (DOCX 16 kb)
