# Supplementary material for: Long-term changes in renal function and perfusion in heart failure patients with reduced ejection fraction
Source: Clin Res Cardiol. 2015 Jun 30;105:10–6. doi: 10.1007/s00392-015-0881-9 (PMC4712227; doi:10.1007/s00392-015-0881-9)
Supplement: Supplementary file 1 — Supplementary material 1 (DOCX 16 kb) [file 392_2015_881_MOESM1_ESM.docx]

## Supplement 1 - Laboratory analyses

NGAL was determined by means of ELISA (R&D systems, Minneapolis, MN). Urine samples were diluted 100 times in 0.1% BSA-PBS buffer. The median coefficient of variation (CV) of the NGAL ELISA was 9.3%. KIM-1 was determined in urine samples by means of ELISA (R&D systems, Minneapolis, MN). Urine samples were diluted two times in 0.1% BSA-PBS buffer. The median CV of the KIM-1 ELISA was 9.0%. NGAL was determined in urine samples by means of ELISA (R&D systems, Minneapolis, MN). Urine samples were diluted 100 times in 0.1% BSA-PBS buffer. The median CV of the NGAL ELISA was 9.3%. NAG was determined by means of a substrate assay in urine samples diluted six times in substrate solution. The enzyme NAG converts substrate p-nitrophenyl N-acetyl-β-D-glucosaminidase at pH 4.5. After 60 minutes at 37°C, 1 M Na_2_CO_3_ was added to the mixture to terminate the reaction and to develop a yellow colour released from the converted substrate. This colour was measured at 400 nm by a microtiter plate reader. The median CV of the NAG assay was 17.5%. Urinary albumin concentrations were determined by nephelometry (Dade Behring Diagnostics, Marburg, Germany) with intra- and inter-assay CV of less than 2.2% and 2.6%, respectively. Urinary albumin excretion was determined as the mean of two 24-h urine collections. Serum and urine creatinine was determined by Kodak Ektachem dry chemistry (Eastman Kodak, Rochester, NY, U.S.A.). The intra- and inter-assay CV were <4.1% and <3.3%, respectively. NT-proBNP was measured by electrochemiluminescence immunoassay on the Roche Elecsys (Roche diagnostics, Netherlands). The intra-assay CV is 1.2 – 1.5%, and the inter-assay CV is 4.4 – 5.0%. High sensitive CRP was determined by nephelometry (BNII N, Dade Behring, Marburg, Germany) with intra- and inter-assay CV than 4.4% and 5.7%, respectively. CRP levels below the detection level were scored as 0.156 mg/l.
